# Stasis ulcer and hydronephrosis after severe genital prolapse: a case report 

**DOI:** 10.1186/s13256-022-03405-8

**Published:** 2022-04-28

**Authors:** Vito Leanza, Alessandra Di Stefano, Erika Carlotta Paladino, Luca Rivoli, Rosario Emanuele Carlo Distefano, Marco Palumbo

**Affiliations:** grid.8158.4 0000 0004 1757 1969 Ist. Patologia Ostetrica e Ginecologica, Department of General Surgery and Medical Surgical Specialities, University of Catania, Via Santa Sofia 78, 95100 Catania, Italy

**Keywords:** Pressure ulcers, Pelvic organ prolapse, Hydronephrosis

## Abstract

**Introduction and importance:**

The most common complication of pelvic organ prolapse is stress urinary incontinence, whereas hydronephrosis or stasis ulcers are quite rare and typical of severe stages. The best treatment for this unusual presentation is still controversial. Here we present our approach.

**Case presentation:**

Here we present the case of a 70-year-old White/Caucasian woman who presented to our hospital with genital procidentia lasting for 10 years that was associated with both hydronephrosis and stasis ulcers.

**Clinical findings and investigations:**

The Pelvic Organ Prolapse Quantitation system was used to assess the severity of the prolapse, being evaluated as stage IV with the apical compartment leading. A thorough search of the literature was conducted to find any similar cases and evaluate the best evidence treatment.

**Interventions and outcomes:**

A no-mesh procedure comprising vaginal hysterectomy, axial apex suspension, and anterior and posterior repair with ulcerated skin removal resulted in complete resolution of both mechanical and functional symptoms. At 3- and 6-month follow-up visits, a satisfying vaginal profile without hydronephrosis was seen. The Pelvic Organ Prolapse Quantitation at 6 months follow-up was as follows: Aa -3, Ba -3, C -7; gh 2, pb 3, tvt 9; Ap -3, Bp -3.

**Relevance and impact:**

Pelvic organ prolapse involves many organs and causes further complications, leading seldom to renal insufficiency and stasis ulcers. A temporary solution to the obstruction can be achieved by utilizing a pessary, but this device cannot be applied when a stasis ulcer has been previously established. The use of mesh for pelvic floor repair is controversial, but a previous vaginal ulcer may represent a contraindication. A complete evaluation and a challenging surgery may allow the recovery of complicated and severe pelvic organ prolapse. Native tissue pelvic repair gives no erosion postsurgical risk, which is the typical complication of the prosthesis.

## Introduction

Pelvic organ prolapse (POP) is displacement of the pelvic organs from their regular position. Rupture or weakness of the pelvic support is the main anatomic cause. POP is divided into anterior compartment prolapses, which can include cystocele and uretrocele, apical compartment prolapses, such as hysterocele, vault, and enterocele, and posterior compartment prolapses, which can be associated with a rectocele. It is often accompanied by urinary, bowel, sexual, psychological, and local pelvic symptoms [1, 2]. Many factors are involved in the pathogenesis, including increasing age, parity, prolonged labor exposure, fetal macrosomia, and delivery modality [3]. The most common complication of POP is stress urinary incontinence, whereas hydronephrosis or stasis ulcers are quite unusual. Hydronephrosis in many cases is a consequence of kinking effect due to obstruction, whereas a stasis ulcer arises from friction of the prolapsed mucosa on the perineal skin. These ulcers are found in women with a severe degree and a history of long-established POP. Venous congestion mechanism which determines edema of tissue results in vaginal fragility and predisposes to tissue alteration. This case is reported in line with the SCARE 2020 criteria [4].

## Case report

### Patient information

A 70-year-old White/Caucasian woman (gravida 6 para 3, 2 spontaneous and 1 vacuum extractor vaginal operative birth) with body mass index (BMI) of 29 kg/m^2^, presented to our unit with genital procidentia lasting for 10 years. During the first vaginal delivery, an episiotomy was performed. She had no significant psychosocial or family medical history. She did not have any previous history of surgeries and did not report any history of traumatic events. She suffered from hypertension, which was treated with ramipril.

She was admitted to the hospital complaining of dysuria, constipation, vaginal pain, and slight bleeding. Her general condition was good, but she was suffering.

Previous blood tests showed an inconstant hypercreatininemia 1.8 mg/dL (normal < 1.3 mg/dL) and hyperuricemia 60 mg/dL (normal< 40 mg/dL), decreasing after a long bed-stay.

### Clinical findings

On inspection, she showed IV-degree cystocele, rectocele, hysterocele, and enterocele associated with both hydronephrosis and stasis ulcers on the exuberant vaginal protruding skin. Vaginal examination revealed complete pelvic bulging with 3 cm ulcerated skin on the posterior compartment, showing the rectal Denonvilliers' fascia. Pelvic Organ Prolapse Quantification (POP-Q) was as follows: Aa +3, Ba +6, C +8; gh 4, pb 1, tvt 9; Ap +3, Bp +6, D +7. Transabdominal and transvaginal ultrasound scans showed uterus of regular size (with atrophic endometrium 1.5 mm), as well as normal ovaries and fallopian tubes, whereas kidneys were hydronephrotic (renal pelvis dilatation was 6 cm on the right and 5 cm on the left side). Postvoiding residual was 365 cc.

### Intervention

After discussing the different options with the patient and acquiring informed consent, we decided to treat all the defects by a vaginal route [5–8]. A no-mesh three-compartmental procedure was carried out by a surgeon experienced in vaginal surgery.

**Fig. 1:**
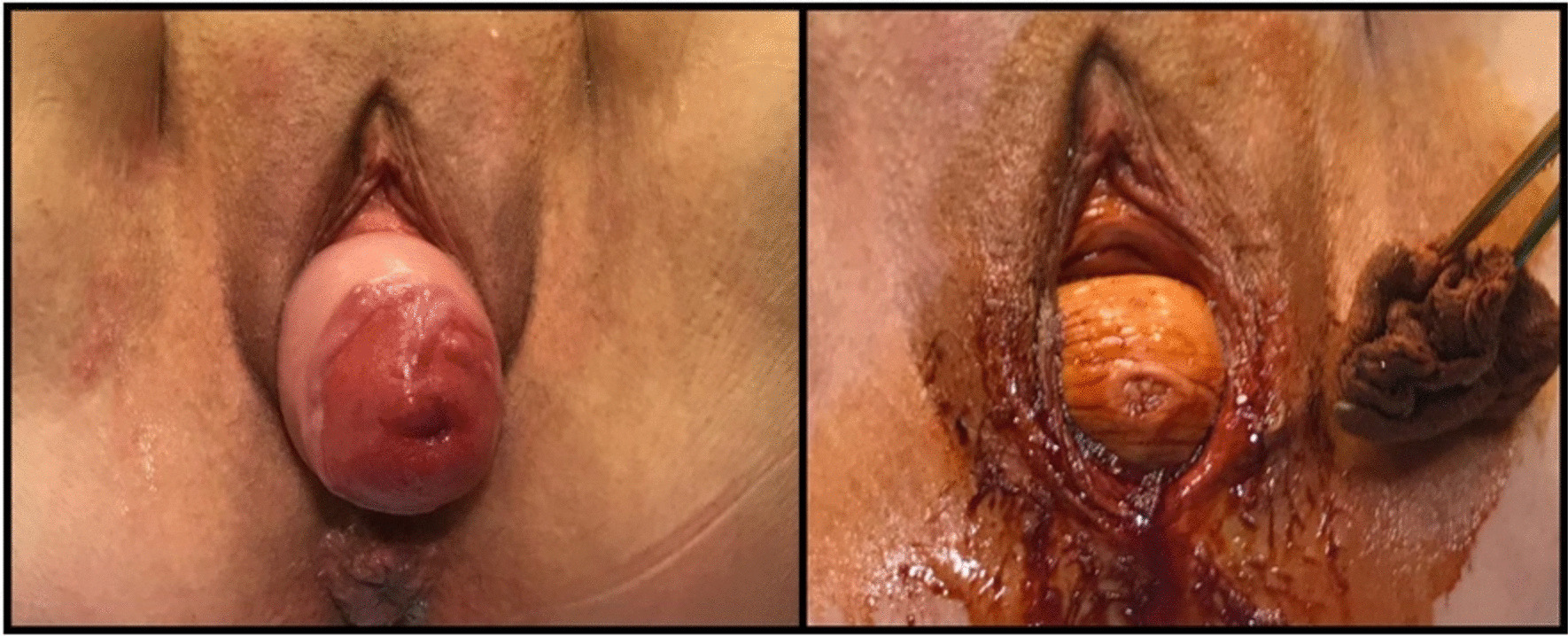
Left: uterine prolapse with cervical erosion; right: posterior compartment stasis ulcer after uterine reduction

**Fig. 2:**
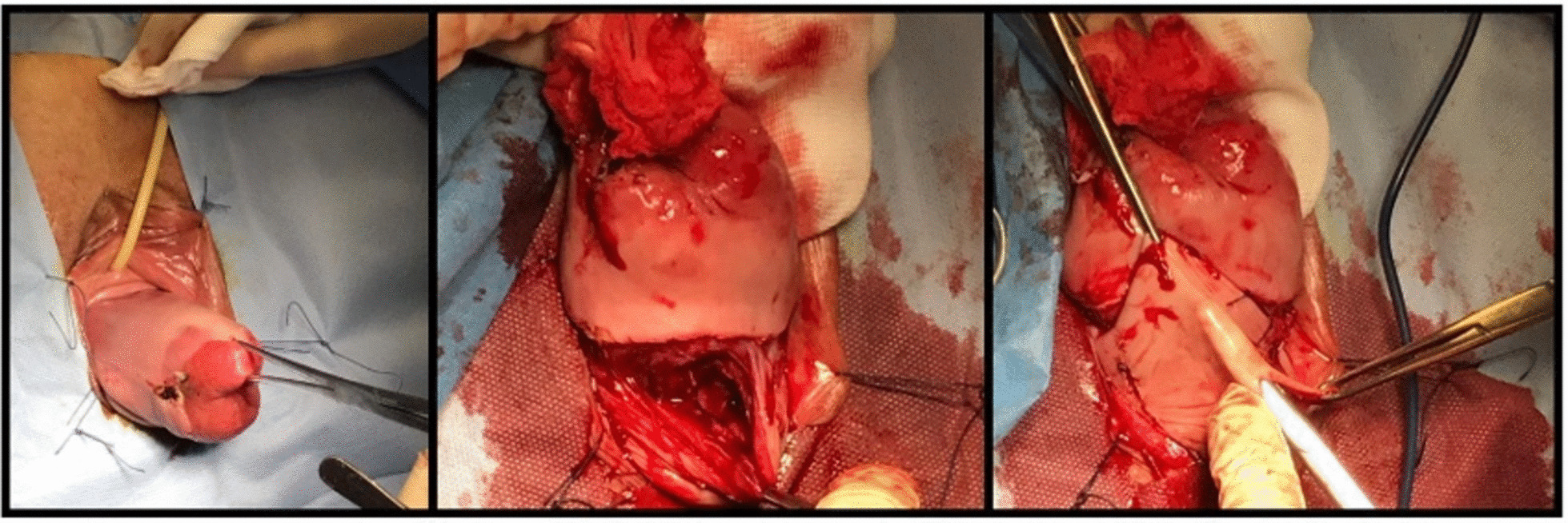
First steps of operation

**Fig. 3:**
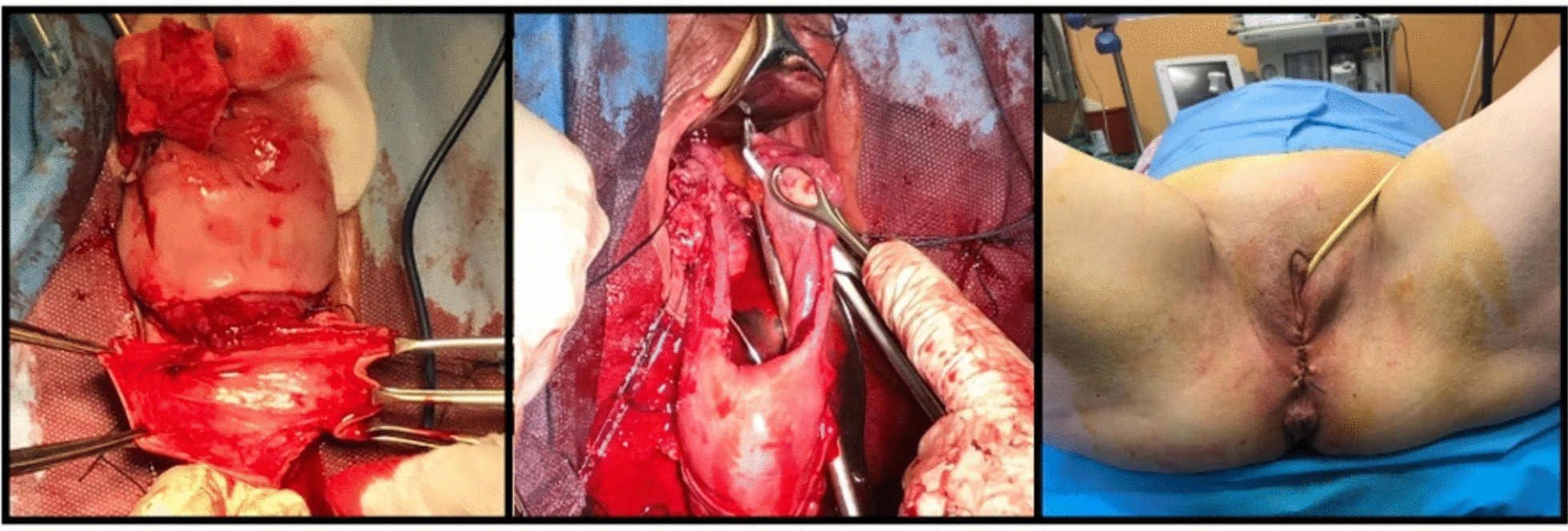
Last steps of operation and final result

After spinal anesthesia, the patient was placed in dorsal lithotomy position, and an indwelling catheter was inserted. Thorough bimanual examination was done before commencing the procedure. Both vulva and vagina were fully prepped with surgical soap solution (Fig. 1). The eroded cervix was exposed by placing a weighted posterior vaginal retractor into the posterior compartment. A small right-angle retractor was used to elevate the anterior vaginal wall; a second right-angle retractor displaced one lateral vaginal wall and exposed the protruding uterus. Two tooth clamps were used to grasp the anterior and posterior lips of the cervix (Fig. 2). The vaginal paracervical mucosa was injected with a physiological solution to obtain separation of tissues. The vaginal mucosa was incised with a scalpel around the entire cervix below the whole erosion (Fig. 2). While traction was applied on the anterior tenacula, dissection of the bladder was done, avoiding bladder damage. With either scissors or sponge-covered fingers, the vesical wall was pushed upward. Strong downward traction was applied to the tenacula on the cervix, and the peritoneum was grasped and incised. By elevating the peritoneal vesicouterine fold with the forceps, the anterior aspect of the uterus was seen. We explored the area by inserting the index finger to be sure of entering anteriorly into the peritoneal cavity and exclude any damage to the bladder. Douglas’ space was also explored. Using two Heaney retractors, the broad ligament was exposed. Placing a finger in the posterior cul-de-sac and moving it laterally revealed the whole uterosacral ligament as it attaches to the posterior aspect of the uterus. A synthetic absorbable suture was used for ligation. Clamping, section, and ligature of paracolpium, parametrium, and uterine vessels were done. Anteroluxation of the uterus was carried out. The round ligament was cut and ligated. After the first ligation, all the ligaments were transfixed again so that the initial and final thread protruded on opposite sides, leaving the needle in site. Infundibulopelvic ligament ligature was performed before removing the uterus with ovaries and fallopian tubes. Employing the needles that were previously left in site, a suture was placed from the tip of uterosacral, cardinal, and round ligament pedicles to the vaginal apex. This procedure obtained hemostasis and physiological suspension of the vagina. The suture was started on the anterior peritoneal edge and brought through all ligaments. The anterior space repair was done as follows: An incision was made along the center of the front wall of the vagina starting near the vaginal entrance and finishing near the top of the vagina. The vaginal mucosa was then separated from the underlying supportive Halban fascial layer. The weakened fascia was then repaired using late absorbable stitches (Fig. 3). Excessive vaginal skin was removed, and the anterior vaginal mucosa was closed with absorbable sutures. The posterior repair was done after removing altered skin and redundant tissue. Perineal suture with perineal muscles juxtaposition ended the procedure.

The bladder catheter was removed within 48 hours, and the patient urinated regularly; the residue was less than 50 cc on the 2nd postoperative course, and in the following three days was absent. Pelvic Floor Impact Questionnaire (PFIQ-7), King’s Health Questionnaire, and locus of control of behavior (LCB) were used to evaluate quality of life. Female Sexual Function Index (FSFI) was evaluated for sexuality determination [6]. The operative course was without complication. Symptoms of frequency, voiding difficulty, and dysuria were improved, gradually. No vaginal pain or bleeding was noted. Six months’ follow-up revealed a satisfying vaginal profile without hydronephrosis. Final POP-Q was: Aa -3, Ba -3, C -7; gh 2, pb 3, tvt 9; Ap -3, Bp -3.

## Discussion

POP involves many organs and causes further complications, leading seldom to renal insufficiency and stasis ulcers [9]. Temporary solution for the obstruction can be achieved by using a pessary, but this device cannot be applied when a stasis ulcer is previously established. In this case, the surgical solution can be performed with or without using mesh. The following points must be evaluated:

- Doctors should consider the patient's history of POP and mechanical aspects.
- It is important to treat predisposing factors such as obesity, obstructive airway disease, constipation, and eventual pelvic masses.
- Although POP can occur in the anterior, middle, or posterior compartments, the pelvic floor should be considered as a single unit, and three-compartmental surgery is mandatory.
- When surgery is needed, doctors should check for potential stress incontinence and, when it is absent, an anti-incontinence procedure is overtreatment and a potentially obstructive surgery.
- A complete evaluation (physical, sexual, and psychological) and adequate treatment is the correct key for recovering from a pelvic disorder [10–14].
- Surgical complications cannot be underestimated, and the jeopardy of fistula remains when stasis ulcers are found [14].

## Conclusions

A challenging surgery may allow the recovery of complicated and severe POP. Absorbable mesh is an attractive choice for augmenting material as it increases strength, but native tissue pelvic repair gives no erosion postsurgical risk, which is the typical complication of the prosthesis.

## Data Availability

All data are included in the medical record of the patient. Clinical data are available from the corresponding author but only on reasonable request.
